# Geroprotective and Radioprotective Activity of Quercetin, (-)-Epicatechin, and Ibuprofen in *Drosophila melanogaster*

**DOI:** 10.3389/fphar.2016.00505

**Published:** 2016-12-23

**Authors:** Ekaterina Proshkina, Ekaterina Lashmanova, Eugenia Dobrovolskaya, Nadezhda Zemskaya, Anna Kudryavtseva, Mikhail Shaposhnikov, Alexey Moskalev

**Affiliations:** ^1^Institute of Biology, Komi Science Center, Ural Branch, Russian Academy of Sciences (RAS)Syktyvkar, Russia; ^2^Laboratory of Genetics of Aging and Longevity, Moscow Institute of Physics and Technology (MIPT)Dolgoprudny, Russia; ^3^Department of Ecology, Institute of Natural Sciences, Syktyvkar State UniversitySyktyvkar, Russia; ^4^Engelhardt Institute of Molecular Biology (EIMB), Russian Academy of Sciences (RAS)Moscow, Russia

**Keywords:** lifespan, stress resistance, *Drosophila melanogaster*, *Caenorhabditis elegans*, quercetin, (-)-epicatechin, ibuprofen

## Abstract

The modulation of longevity genes and aging-associated signaling pathways using pharmacological agents is one of the potential ways to prolong the lifespan and increase the vitality of an organism. Phytochemicals flavonoids and non-steroidal anti-inflammatory drugs have a large potential as geroprotectors. The goal of the present study was to investigate the effects of long-term and short-term consumption of quercetin, (-)-epicatechin, and ibuprofen on the lifespan, resistance to stress factors (paraquat, hyperthermia, γ-radiation, and starvation), as well as age-dependent physiological parameters (locomotor activity and fecundity) of *Drosophila melanogaster*. The long-term treatment with quercetin and (-)-epicatechin didn't change or decreased the lifespan of males and females. In contrast, the short-term treatment with flavonoids had a beneficial effect and stimulated the resistance to paraquat and acute γ-irradiation. The short-term ibuprofen consumption had a positive effect on the lifespan of females when it was carried out at the middle age (30–40 days), and to the survival of flies under conditions of oxidative and genotoxic stresses. However, it didn't change the lifespan of males and females after the treatment during first 10 days of an imago life. Additionally, quercetin, (-)-epicatechin, and ibuprofen decreased the spontaneous locomotor activity of males, but had no effect of stimulated the physical activity and fecundity of females. Revealed quercetin, (-)-epicatechin, and ibuprofen activity can be associated with the stimulation of stress response mechanisms through the activation of pro-longevity pathways, or the induction of hormesis.

## Introduction

The modulation of longevity genes and aging-associated signaling pathways using chemicals is one of the potential ways to increase the human lifespan. Recently, more attention was given to natural compounds as they are considered safer for use (Carretero et al., 2015). The geroprotective effects of a variety of phytochemicals including flavonoids are already revealed (Leonov et al., 2015). At the same time, the most of age-related pathologies and aging process are associated with chronic inflammation (Franceschi and Campisi, 2014). Therefore, inhibition of this process using anti-inflammatory drugs can be an effective geroprotective method.

Flavonoids is a class of polyphenolic natural chemicals with revealed antioxidant, anticancer, antidiabetic, cardioprotective, and neuroprotective properties (Zern and Fernandez, 2005; Seelinger et al., 2008; Vauzour et al., 2008; Ruijters et al., 2013; Vinayagam and Xu, 2015). However, their ability to demonstrate positive effects is highly dependent on applied concentrations. In high concentrations, even toxic effects can be observed. For example, flavonoid (−)-epigallocatechin 3-gallate in low concentrations (1–10 μM) protects neuronal cells against oxidative damage (Levites et al., 2002). In contrast, pro-oxidant properties of the compound were observed, when higher concentrations were used (10–50 μM). Quercetin and (-)-epicatechin are among the most well-studied flavonoids. They are abundant in the human diet (Arts et al., 2000a,b; Boots et al., 2008). Quercetin belongs to a subclass of flavonols and is mainly consumed by humans with onions and tea (Hertog et al., 1993). (-)-epicatechin belongs to flavanols and can be mainly found in cocoa and green tea (Shay et al., 2015). The health-benefiting properties of quercetin and (-)-epicatechin are determined by their antioxidant and/or non-antioxidant activity. The direct antioxidant activity is their ability to scavenge reactive oxygen and nitrogen species (Jung et al., 2003). The non-antioxidant activity is expressed in the modulation of different molecular processes including anti-inflammatory (Nair et al., 2006; Vicentini et al., 2011), anti-cancer (Granado-Serrano et al., 2006) activities, regulation of several aging-associated molecular pathways (Granado-Serrano et al., 2006, 2010), and others.

Effects of non-steroidal anti-inflammatory drugs are dose-dependent as well (Danilov et al., 2015). Their intake at low concentrations extended the *Drosophila melanogaster* lifespan and survival under stress conditions, and delayed the age-dependent decline of locomotor activity. Recently, ibuprofen was characterized as a geroprotector on the several experimental models including *Saccharomyces cerevisiae, Caenorhabditis elegans*, and *Drosophila melanogaster* (He et al., 2014). It was shown that ibuprofen extends the replicative lifespan of yeast cells by the decreasing of tryptophan intake, and causes the reducing of cellular size at birth and the proliferation delay. However, there are no complex investigations of their influence on the age-depending physiological parameters and the resistance of an organism to different kinds of stress factors.

The goal of the present study was to investigate the effects of long-term and short-term consumption of quercetin, (-)-epicatechin, and ibuprofen on the lifespan, stress resistance (oxidative, thermal, genotoxic stresses, and satravation), spontaneous locomotor activity and fecundity of *Drosophila melanogaster*. Obtained results demonstrated that the long-term of quercetin and (-)-epicatechin treatment didn't significantly influence the lifespan of males and females or had a negative action. In contrast, the short-term treatment with flavonoids had a beneficial effect and stimulated the resistance to paraquat and acute γ-irradiation. A life extending effect was also shown in females under conditions of short-term ibuprofen consumption at the middle age (30–40 days), but not at the young age (0–10 days). Additionally, ibuprofen had a frank positive effect to the survival of flies under oxidative and genotoxic stresses. We obtained converse effects of these pharmacological agents to the physiological parameters in males and females. Quercetin, (-)-epicatechin, and ibuprofen decreased the spontaneous locomotor activity of males, but had no effect or stimulated the physical activity and fecundity of females.

## Materials and methods

### *Drosophila melanogaster* strain and maintenance conditions

Wild-type *Canton-S* flies (Bloomington Stock Center, Bloomington, USA) were used in the experiment. Animals were housed in 25 ml vials containing 5 ml of the nutrient medium with the following composition (for 1 liter of water): dry yeast, 8 g; agar, 7 g; sugar, 30 g; semolina, 30 g; propionic acid, 8 drops (Ashburner, 1989). Standard conditions were maintained (25°C, 60% relative humidity; 12 h light/dark regime).

Experimental animals were produced by breeding, which involved transferring 10–15 pairs of males and females into vials with the nutrient medium and allowing them to stand for 24 h for oviposition. After imago hatching, flies were anastezied using CO_2_, separated by sex, and transferred in vials containing the nutrient medium with investigated drugs (30 flies per vial).

In the experiments, we used physiological concentrations of quercetin, (-)-epicatechin, and ibuprofen (Sigma, USA). Drugs were dissolved in 98% ethanol in concentrations 30, 50, and 100 μM. These solutions were added to the yeast paste in the proportion 1:100 and spread on the surface of the nutrient medium. For control flies, the same amount of ethanol without chemicals was applied.

### Lifespan analysis

To determine the effect of studied drugs on longevity, 100–200 flies were collected for each experimental group (30 flies per vial). Males and females were analyzed separately. For the analysis of the influence of the long-term intake of investigated drugs, flies were kept on the nutrient medium spread with the yeast past containing chemicals during lifetime (from imago hatching to death). For the detection of short-term treatment effects, drugs were added during first 10 days of an imago life, as well as at the age of 30–40 days. Then flies were transferred in vials with the standard medium.

Flies were transferred to the fresh medium twice a week. Every day the number of dead animals was counted. The survival functions were calculated using Kaplan–Meier method and displayed as survival curves (Kaplan and Meier, 1992). The mean, median, minimum, maximum lifespans, the age of 90% mortality and other parameters were counted. The nonparametric Kolmogorov-Smirnov (Fleming et al., 1980), Cox-Mantel (Mantel, 1966), Gehan-Breslow-Wilcoxon (Breslow and Zandstra, 1970) tests were used to estimation the significance of differences in survival data between samples. The statistical significance between maximum lifespan values was assessed using the Wang-Allison method (Wang et al., 2004). The α and R_0_ parameters of the Gompertz equation [μ(x) = R_0_ e^αx^] and mortality rate doubling time (MRDT = ln2/α) were also calculated (Finch, 1990). The statistical data analysis was performed using Statistica, version 6.1 (StatSoft, USA) and software environment R, version 2.15.1 (R core Team). Each experiment was performed in two independent replications.

### Fecundity assay

For each variant of the experiment, 50 females were selected and putted separately into the vials with the nutrient colored with activated carbon. Into each vial was added one *Canton-S* male, which was not treated with tested substances. All flies were maintained under same conditions and transferred to the fresh medium once a week. Old males were replaced with young flies. Once a week, the number of eggs laid by the females during 24 h was counted. The statistical significance between samples was estimated using the χ^2^ test (Fisher, 1954). The statistical data analysis was performed using the program Statistica, version 6.1 (StatSoft, USA).

### *Drosophila melanogaster* locomotor activity assay

For each experimental variant, 30 flies were collected (10 flies per vial). Males and females were analyzed separately. Flies were transferred to the fresh medium once a week. Spontaneous locomotor activity was tested every week using the hardware-software complex “*Drosophila* Population Monitor” (TriKinetics Inc., USA). Data were collected during 24 h, and the statistical analysis was performed using the χ^2^ test by the program Statistica, version 6.1 (StatSoft, USA).

### Stress resistance analysis

To measure the effects of investigated chemicals on the stress resistance, 100–150 flies were collected for each experimental group (30 flies per vial). Males and females were analyzed separately. Flies were placed in conditions of an intensive stress from 10th day after imago hatching and treatment with investigated drugs. In the test to oxidative stress resistance, flies were transferred into vials with the filter paper soaked with 200 ml of the 20 mM paraquat (Methyl viologen dichloride hydrate, Sigma, USA) solution in 5% sucrose. To measure the resistance to hyperthermia, flies were kept in vials with the standard nutrient medium at 35°C. To determine the resistance to starvation, flies were placed in vials with 2% agar as a medium. The number of dead flies was counted twice a day. Statistical analysis of survival data were performed as in the case with lifespan analysis.

### Radioresistance assay

To determine the effect of pharmacological agents to radioresistance, 200–300 flies were collected for each variant of the experiment (30 flies per vial). Males and females were analyzed separately. Flies were kept in vials with the nutrient medium spread with the yeast past containing drugs in the concentration 1.0 μM during 10 days. Then flies were exposed by the acute (22 h) γ-irradiation from the source with ^60^Co at the accumulated dose 1000 Gy. Irradiated animals were transferred to standard conditions, and the number of dead flies was counted every 24 h. Statistical analysis of survival data were performed using the χ^2^ test by the program Statistica, version 6.1 (StatSoft, USA).

### Quantitative real-time PCR analysis

To identify molecular and genetic mechanisms involved in the observed effects, expression changes in stress-response genes were analyzed. These included genes that encode proteins involved in the detoxification of reactive oxygen species (*Sod1*), heat shock response (*Hsp70*), regulation of the response to DNA damages (*Gadd45*), DNA excision repair (*Mus210-*homologs *XPC*), and double-strand break repair (*Spn-B*-homologs *XRCC3*). Quantitative real-time RT-PCR analysis was performed on the 10th day of the consumption of drugs in the concentration 0.3 μM.

For each experimental condition, 15 flies were used. The levels of gene expression in males and females samples were evaluated separately. RNA was isolated using Aurum Total RNA Mini Kit (Bio-Rad) accordingly to the manufacturer's instructions. The reverse transcription for cDNA synthesis was performed using iScript cDNA Synthesis Kit (Bio-Rad) in accordance with the manufacturer's protocol. The reaction mixture for real-time PCR contained iTaq Universal SYBR Green Supermix (Bio-Rad) and primers of studded genes and reference gene (β-*Tubulin*) (Syntol) (Table 1). This mixture was pipetted in a volume 8 μl into plates' wells with 200 μl capacity. Then 2 μl of cDNA for each sample was added.

**Table 1:** **Primers for real-time RT-PCR**.

**Target**	**Forward primer**	**Reverse primer**
*β-Tubulin*	5′-GCAACTCCACTGCCATCC-3′	5′-CCTGCTCCTCCTCGAACT-3′
*Sod1*	5′-TGCACGAGTTCGGTGACAACAC-3′	5′-TCCTTGCCATACGGATTGAAGTGC-3′
*Gadd45*	5′-GCAAACGCACAACCAAAC-3′	5′-GGCCATCAGGCAGAAGAG-3′
*Mus210*	5′-AGAAGACGGTGCATTTGAGATTGC-3′	5′-CCTCGCAAACAATGAAGCCATCG-3′
*Spn-B*	5′-AGATTGCTGCAGATGAGCAAAGCC-3′	5′-TTTATAACGCACGCCAGGAGAGGT-3′
*Hsp70*	5′-TCCTCAGCGGAGACCAGA-3′	5′-CACGTTCGCCCTCATACA-3′

The reaction was performed in a CFX96 amplifier (BioRad) using a following program: (1) denaturation for 10 min at 95°C, (2) denaturation for 15 s at 95°C, (3) annealing for 30 s at 60°C, (4) elongation for 30 s at 60°C. Steps 2–4 were repeated 50 times. The expression of the studied genes was normalized using the β*-Tubulin* housekeeping gene. Their amplification was performed in separate tubes. The relative levels of expression were calculated using CFX96 Software (BioRad). The statistical significance of differences between samples were evaluated using Mann-Whitney *U*-test by the program Statistica, version 6.1 (StatSoft, USA). The experiment for each variant of drugs' treatment was made in triplicates.

## Results

### Effects of long-term and short-term quercetin, (-)-epicatechin, and ibuprofen treatment on the *Drosophila melanogaster* lifespan

**Figure 1:**
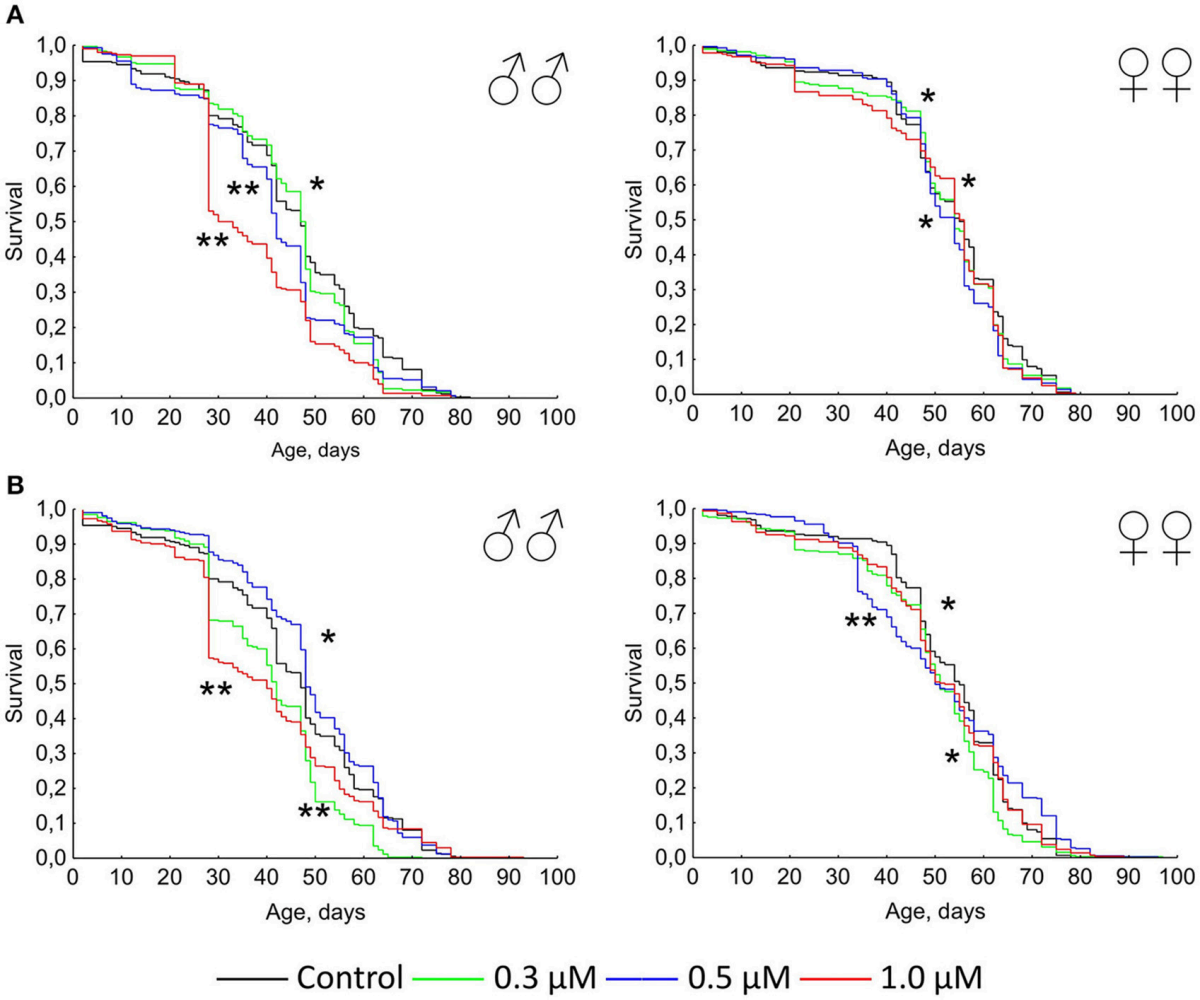
**Survivorship curves of ***Drosophila melanogaster*** males (♂♂) and females (♀♀) treated with quercetin (A)** and (-)-epicatechin **(B)** during lifetime (combined data of two independent replications); ^*^p < 0.05, ^**^p < 0.001, Kolmogorov-Smirnov test.

Previously it was shown that quercetin and (-)-epicatechin increase the lifespan of different model organisms including nematode *Caenorhabditis elegans*, fruit fly *Drosophila melanogaster* and mouse *Mus musculus* (Pietsch et al., 2009; Si et al., 2011). However, in the most cases we didn't found the frank life extending effect of these two flavonoids under conditions of their long-term application on flies. In contrast, the treatment with 0.3–1.0 μM of quercetin during *Drosophila melanogaster* lifetime decreased the median and maximum lifespan of males and females by 2–33 and 4–13% (*p* < 0.05), respectively (Table 2, Figure 1A). The same longevity decline was detected for 0.3–1.0 μM (-)-epicatechin which reduced the fruit fly median and maximum lifespan by 6–15 and 6–15% (*p* < 0.05), respectively (Table 2, Figure 1B).

**Table 2:** **Lifespan parameters of ***Drosophila melanogaster*** treated with quercetin and (-)-epicatechin during lifetime**.

**Treatment**	**Concentration**	**Min**	**$\bar{X}\pm \Delta m$**	***M***	**90%**	**Max**	**MRDT**	**α**	***R_*0*_***	***N***
**MALES**										
Control (1)		2	40.5 ± 1.3	47	58	76	9.6	0.076	0.0024	153
Quercetin (1)	0.3 μM	2	40.1 ± 1.5^*^	47	49^**^	55	5.6	0.123	0.0004	92
	0.5 μM	2	32.4 ± 1.7^**^	41^**^	48^**^	55	10.7	0.065	0.0055	97
	1.0 μM	2	41.7 ± 1.3	47	49^*^	63	6.1	0.114	0.0006	92
(-)-Epicatechin (1)	0.3 μM	2	43.8 ± 1.0	48	55	72	7.0	0.099	0.0008	144
	0.5 μM	6	44.7 ± 1.4^*^	47	66^**^	68	9.5	0.073	0.0017	127
	1.0 μM	2	37.9 ± 1.3^*^	42	55^*^	62	8.7	0.079	0.0024	150
Control (2)		2	48.7 ± 1.3	48	72	82	11.4	0.061	0.0020	193
Quercetin (2)	0.3 μM	5	46.6 ± 1.0^*^	48	64^**^	78	9.8	0.071	0.0016	212
	0.5 μM	13	46.1 ± 1.1^*^	42^*^	64	79	11.2	0.062	0.0024	193
	1.0 μM	2	35.2 ± 1.0^**^	28^**^	62^**^	78	14.6	0.048	0.0077	208
(-)-Epicatechin (2)	0.3 μM	2	37.3 ± 1.0^**^	35^**^	62^**^	64	11.0	0.063	0.0043	196
	0.5 μM	2	50.7 ± 1.1	50	68	78	9.3	0.074	0.0010	191
	1.0 μM	2	41.1 ± 1.5	28^**^	72	93	19.4	0.036	0.0074	183
**FEMALES**										
Control (1)		2	46.9 ± 1.3	48	62	82	9.0	0.078	0.0013	134
Quercetin (1)	0.3 μM	2	41.3 ± 1.6^**^	47^*^	55^*^	65	7.9	0.088	0.0014	86
	0.5 μM	2	44.4 ± 1.5^*^	48	61	68	7.6	0.091	0.0009	86
	1.0 μM	2	35.9 ± 1.7^**^	40^**^	55^*^	62	10.9	0.064	0.0045	90
(-)-Epicatechin (1)	0.3 μM	2	43.4 ± 1.5	49	62	79	10.4	0.067	0.0023	131
	0.5 μM	5	41.1 ± 0.8^**^	38^**^	59	76	9.7	0.071	0.0026	219
	1.0 μM	2	42.0 ± 1.4^*^	47^*^	56^*^	93	11.3	0.061	0.0033	129
Control (2)		2	56.4 ± 1.0	58	72	78	7.3	0.096	0.0003	180
Quercetin (2)	0.3 μM	2	55.9 ± 1.0	57	68	78	6.9	0.100	0.0002	190
	0.5 μM	7	54.2 ± 0.9^*^	56^*^	64^*^	78	7.0	0.099	0.0003	194
	1.0 μM	2	56.8 ± 0.9	57	68	79	5.9	0.118	0.0001	188
(-)-Epicatechin (2)	0.3 μM	2	52.3 ± 1.0^**^	54^**^	65^*^	97	9.2	0.075	0.0009	199
	0.5 μM	2	61.8 ± 1.0^**^	64^**^	78^**^	96	8.2	0.084	0.0003	206
	1.0 μM	2	57.4 ± 1.1	62	72	89	8.1	0.086	0.0004	166

Min, minimum lifespan, days; $\bar{X}\pm \Delta m$, mean lifespan and standard error of mean, days; M, median lifespan, days; 90%, age of 90% mortality, days; Max, maximum lifespan, days; MRTD, mortality rate time of doubling; α and R_0_, Gompertz equation parameters; N, number of flies in a sample; (1), (2), replications;

* *p < 0.05*,

** *p < 0.001, data in forth column calculated by Cox–Mantel test, in fifth column calculated by Gehan-Breslow-Wilcoxon test, in sixth column calculated by Wang–Allison method*.

Recently, it was found that non-steroidal anti-inflammatory drug ibuprofen increases the lifespan of *Saccharomyces cerevisiae, Caenorhabditis elegans*, and *Drosophila melanogaster* (He et al., 2014). For fruit flies treated with 0.3–1.0 μM of ibuprofen, the increase of median lifespan was 6–17% (*p* < 0.05). Therefore, we compared other investigated parameters of quercetin and (-)-epicatechin treatment with ibuprofen to reveal their geroprotective, adaptogenic, radioprotective activities.

**Figure 2:**
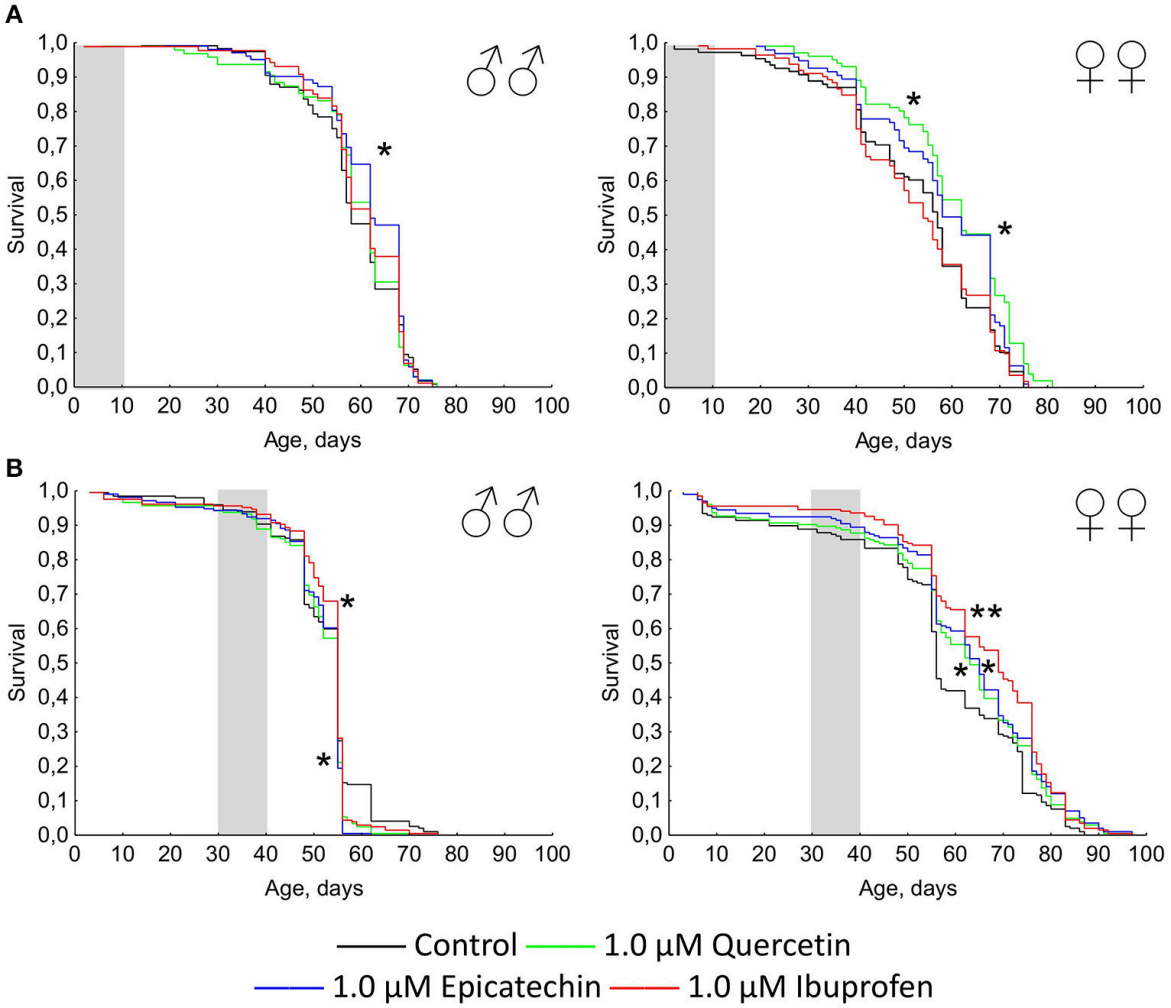
**Survivorship curves of ***Drosophila melanogaster*** males (♂♂) and females (♀♀) treated with quercetin, (-)-epicatechin, and ibuprofen during 10 days at the age of 0–10 days after imago hatching (A)** and at the age of 30–40 days **(B)**; ^*^p < 0.05, ^**^p < 0.001, Kolmogorov-Smirnov test.

The short-term treatment during first 10 days of the lifetime with quercetin and (-)-epicatechin has a favorable effect on the *Drosophila* longevity (Figure 2A). The median and maximum lifespan of females was increased by 13 and 6% (*p* < 0.05), respectively, after the intake of 1.0 μM quercetin. (-)-epicatechin at the concentration 1.0 μM extended the median lifespan of *Drosophila* males and females by 7–8% (*p* < 0.05). However, the treatment during 10 days with ibuprofen was too insufficient for pro-longevity effect.

A positive action of the short-term treatment at the age of 30–40 days with investigated drugs was detected for females, but not for males (Figure 2B). The median lifespan of *Drosophila* females was extended by 13, 16, and 23% after the addition of 1 μM quercetin, (-)-epicatechin, and ibuprofen, respectively (*p* < 0.05).

Thus, the geroprotective effect was found for the short-term application of quercetin and (-)-epicatechin, and it was absented for the long-term eating of these drugs. At the same time, ibuprofen didn't influence the lifespan when it was used in young flies, but have a benefit effect after the treatment of middle-age females during 10 days.

### The influence of quercetin, (-)-epicatechin, and ibuprofen on the *Drosophila melanogaster* fecundity and locomotor activity

**Figure 3:**
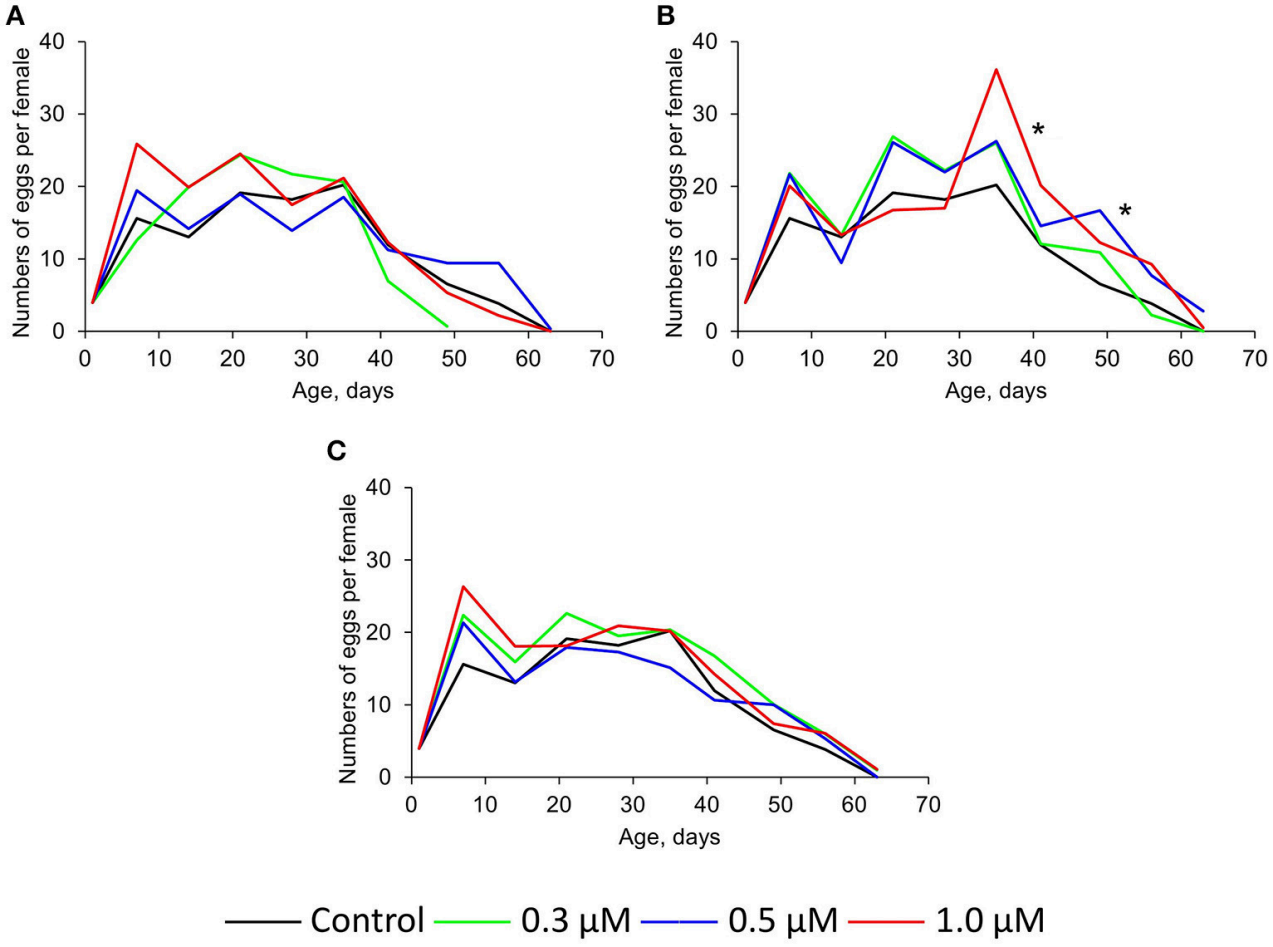
**Fecundity of ***Drosophila melanogaster*** females treated with quercetin (A)**, (-)-epicatechin **(B)**, and ibuprofen **(C)**, ^*^p < 0.001, χ^2^-test.

The intake of 0.3–1.0 μM of quercetin and ibuprofen didn't sufficiently change the fecundity of *Drosophila* females compared with control group (Figures 3A,C). At the same time, (-)-epicatechin at concentrations 0.5 and 1.0 μM increased this physiological parameter by 33–34% (*p* < 0.001) on average (Figure 3B).

**Figure 4:**
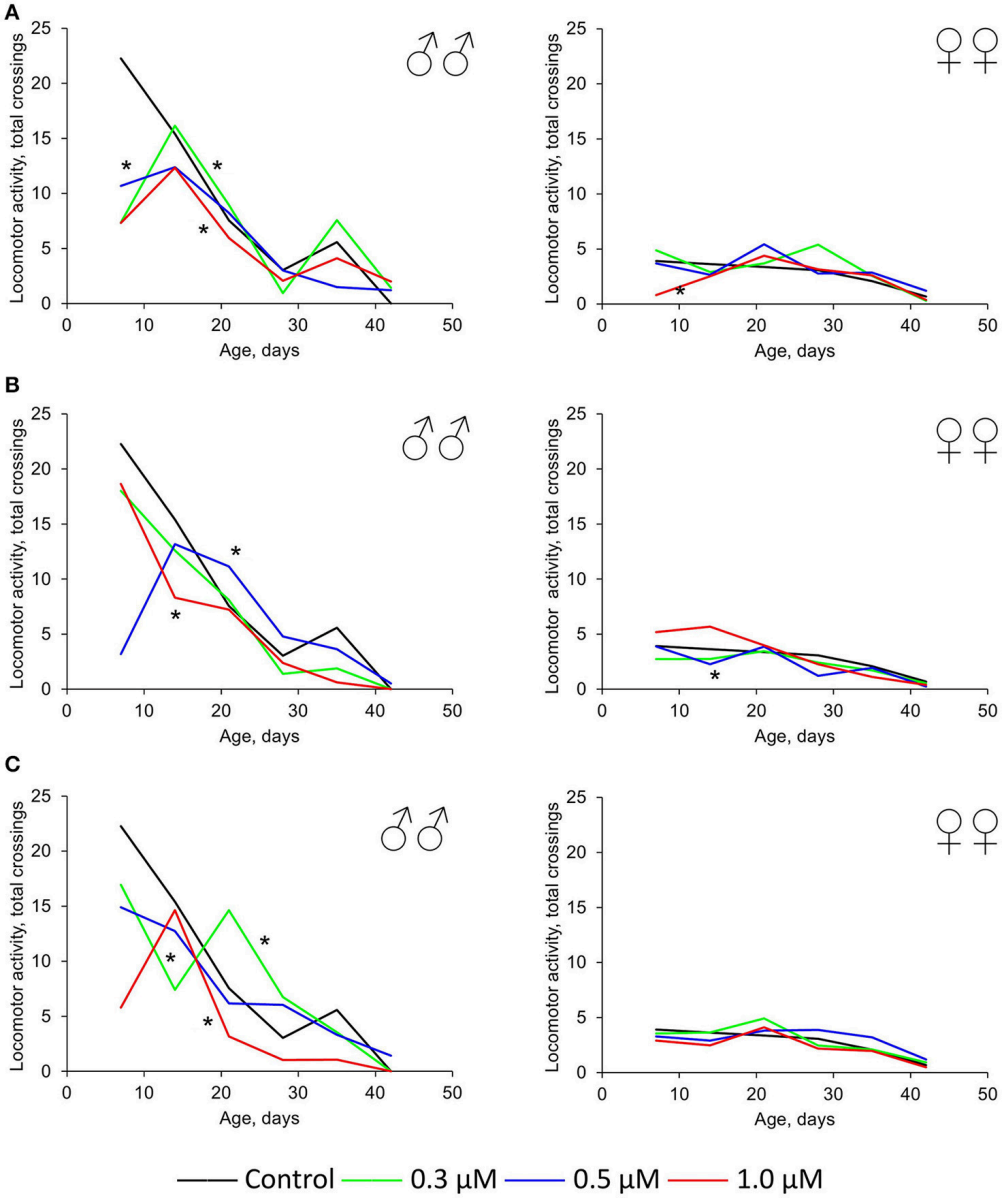
**Spontaneous locomotor activity of ***Drosophila melanogaster*** males (♂♂) and females (♀♀) treated with quercetin (A)** , (-)-epicatechin **(B)**, and ibuprofen **(C)**; ^*^p < 0.001, χ^2^-test.

In females, the neutral or positive effect (by 2.5–2.1 times) of these three drugs was also detected for spontaneous locomotor activity. In males, this parameter was higher compared with females under both control and experimental conditions. Nevertheless, the intake of quercetin, (-)-epicatechin, and ibuprofen led to the physical activity decrease of *Drosophila* males by 18–62% (*p* < 0.001) on average (Figure 4). The most frank negative effect was detected at time points 1 week and 5–6 weeks. In this way, the suppressive impact of these drugs strengthens with age.

### Stress-resistance changes after quercetin, (-)-epicatechin, and ibuprofen treatment

**Figure 5:**
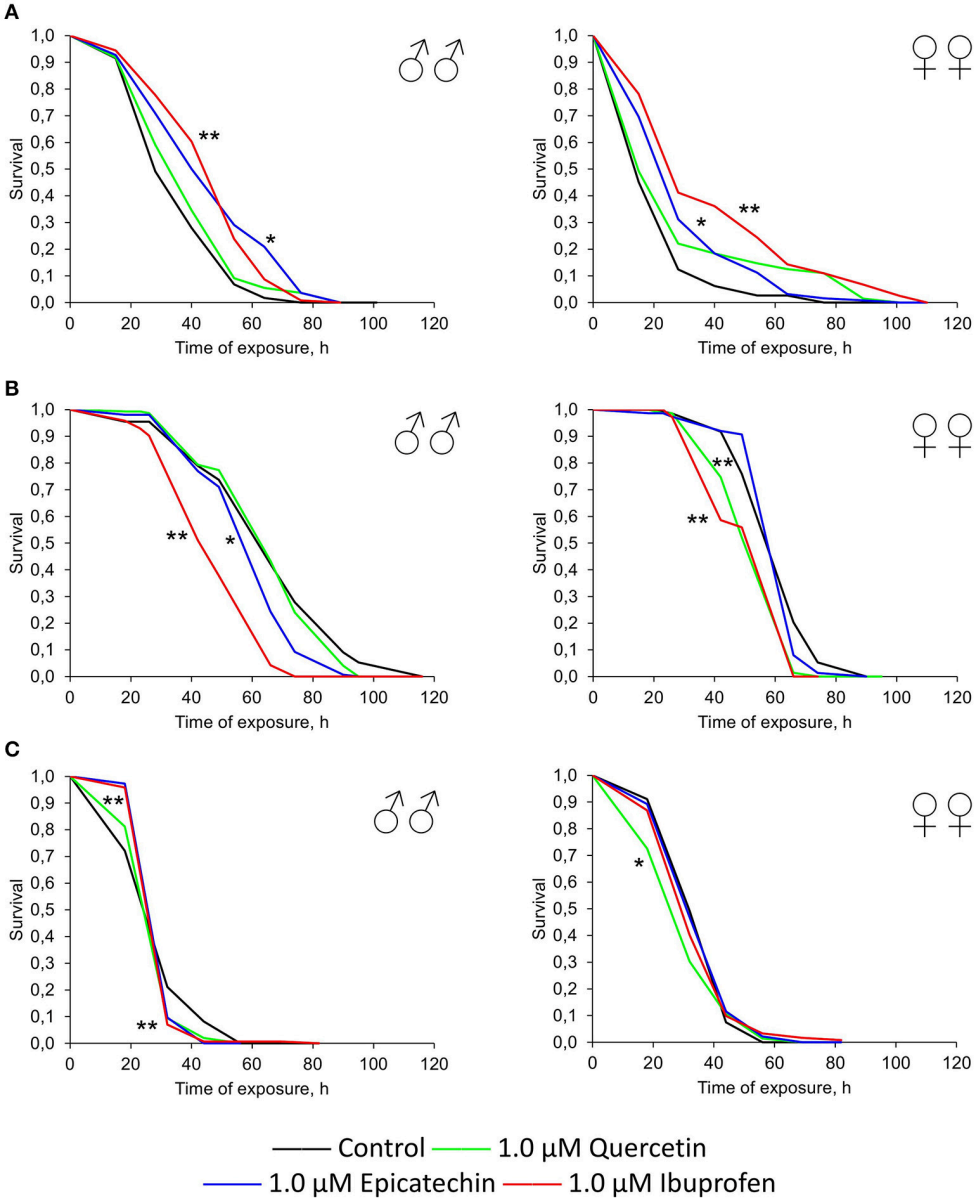
**Resistance to oxidative stress (20 mM paraquat) (A)**, heat shock (35°C) **(B)** and starvation **(C)** of *Drosophila melanogaster* males (♂♂) and females (♀♀) treated with quercetin, (-)-epicatechin, and ibuprofen; ^*^p < 0.05, ^**^p < 0.001, Kolmogorov-Smirnov test.

To reveal the influence of quercetin, (-)-epicatechin, and ibuprofen on the stress resistance we analyzed the survival of flies previously treated with these drugs under conditions of oxidative stress (20 mM paraquat), hyperthermia (35°C), and starvation. In the most cases, (-)-epicatechin and ibuprofen (but not quercetin) increased the resistance of fruit flies to the inductor of free radicals paraquat. The mean survival of males and females that ate nutrient with 0.5–1.0 μM (-)-epicatechin and 0.3–1.0 μM ibuprofen was 11–43% higher (*p* < 0.05) compared with control animals (Table 3, Figure 5A). The most positive effect was found for the anti-inflammatory drug ibuprofen.

**Table 3:** **Survival parameters of ***Drosophila melanogaster*** treated with quercetin, (-)-epicatechin, and ibuprofen under conditions of oxidative stress (20 mM paraquat), hyperthermia (35°C) and starvation**.

**Treatment**	**Concentration**	**Gender**							
		**Males**				**Females**			
		**$\bar{X}\pm \Delta m$**	***M***	**90%**	***N***	**$\bar{X}\pm \Delta m$**	***M***	**90%**	***N***
**20 mM PARAQUAT**									
Control		37.6 ± 1.3	28	54	118	23.8 ± 1.2	15	40	113
Quercetin	0.3 μM	41.3 ± 1.3	40^*^	54	118	23.3 ± 1.0	15	40	120
	0.5 μM	42.7 ± 1.4^*^	40^*^	54	116	24.1 ± 1.0	28	40	116
	1.0 μM	40.9 ± 1.6	40	54	110	31.2 ± 2.1^*^	15	89^*^	136
(-)-Epicatechin	0.3 μM	38.1 ± 1.4	40	54	111	24.9 ± 1.2	15	52	123
	0.5 μM	43.2 ± 1.6^*^	40^*^	64	119	34.6 ± 2.0^**^	28^**^	64^*^	118
	1.0 μM	48.5 ± 1.9 ^**^	47^**^	76^*^	110	32.2 ± 1.6^**^	28^**^	64^*^	125
Ibuprofen	0.3 μM	44.6 ± 1.9^**^	40^*^	64^*^	107	26.7 ± 1.5	28	54	117
	0.5 μM	43.6 ± 1.6^*^	40^*^	64	109	36.1 ± 1.7^**^	28 ^**^	64^*^	127
	1.0 μM	48.6 ± 1.5^**^	54^**^	64^*^	126	41.8 ± 2.4^**^	28^**^	89^*^	119
**35**°**C HYPERTHERMIA**									
Control		68.3 ± 1.9	66	90	133	63.5 ± 1.0	66	74	133
Quercetin	0.3 μM	70.9 ± 1.5	66	90	136	58.0 ± 0.9^**^	66^**^	66^**^	132
	0.5 μM	63.6 ± 1.4^*^	66^*^	90^*^	136	68.0 ± 1.0 ^*^	66^*^	90^*^	130
	1.0 μM	67.9 ± 1.4	66	90	150	55.9 ± 0.9^**^	66^**^	66^*^	146
(-)-Epicatechin	0.3 μM	57.6 ± 1.3^**^	66^**^	74^**^	152	66.6 ± 0.7	66^*^	74	141
	0.5 μM	69.9 ± 1.5	66	90^*^	135	57.1 ± 1.0^**^	66^**^	66^*^	119
	1.0 μM	62.4 ± 1.2^**^	66^*^	74^**^	152	64.2 ± 1.1	66	66	75
Ibuprofen	0.3 μM	60.9 ± 1.5^**^	66^*^	90^*^	163	52.9 ± 1.2^**^	49^**^	66^**^	138
	0.5 μM	52.0 ± 1.2^**^	45.5^**^	66^**^	154	59.3 ± 0.9 ^**^	66^**^	66^*^	145
	1.0 μM	50.3 ± 1.2^**^	49^**^	66^**^	143	55.2 ± 1.2^**^	66^**^	66^*^	111
**STARVATION**									
Control		31.6 ± 0.9	32	44	147	49.8 ± 0.8	44	56	134
Quercetin	0.3 μM	29.2 ± 0.6^*^	32	32^*^	151	47.6 ± 0.8	44^*^	56	157
	0.5 μM	31.1 ± 0.5	32	32^**^	147	51.0 ± 0.9	56	69	148
	1.0 μM	30.7 ± 0.6	32	32^*^	149	45.9 ± 1.0 ^*^	44^**^	68	142
(-)-Epicatechin	0.3 μM	29.6 ± 0.6	32	32^*^	124	45.5 ± 0.8^**^	44^**^	56	153
	0.5 μM	29.7 ± 0.5^*^	32	32^**^	155	41.6 ± 0.9^**^	44^**^	56	149
	1.0 μM	32.8 ± 0.4	32^*^	32^**^	145	49.9 ± 0.9	44	69	140
Ibuprofen	0.3 μM	31.8 ± 0.4	32	32^**^	152	47.5 ± 0.9	44^*^	56	150
	0.5 μM	31.1 ± 0.5	32	32^*^	149	50.9 ± 0.9	56	69	151
	1.0 μM	32.5 ± 0.5	32	32^*^	143	49.2 ± 1.1	44	56	122

$\bar{X}\pm \Delta m$, mean survival and standard error of mean, h; M, median survival, h; 90%, age of 90% mortality, h; N, number of flies in a sample;

* *p < 0.05*,

** *p < 0.001, data in third and seventh columns calculated by Cox–Mantel test, in fourth and eighth columns calculated by Gehan-Breslow-Wilcoxon test, in fifth and ninth columns calculated by Wang–Allison method*.

At the same time, quercetin, (-)-epicatechin, and ibuprofen didn't significant change or decrease the survival of males and females under the hyperthermia and starvation in the most cases (Table 3, Figures 5B,C).

**Figure 6:**
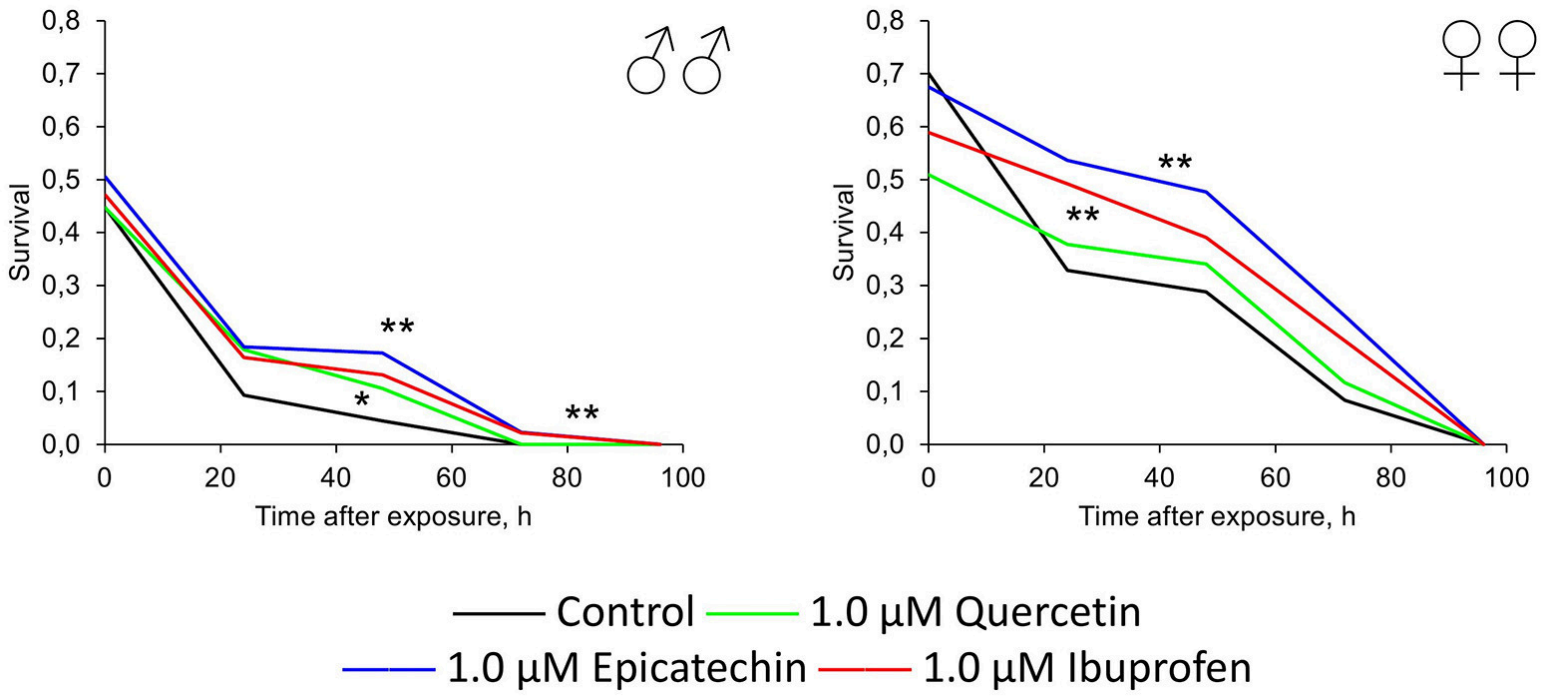
Resistance to acute γ-irradiation (1000 Gy) of ***Drosophila melanogaster*** males (♂♂) and females (♀♀) treated with quercetin, (-)-epicatechin, and ibuprofen ^*^p < 0.05, ^**^p < 0.001, χ^2^-test.

In addition to the effect of quercetin, (-)-epicatechin, and ibuprofen on the resistance of flies to oxidative stress, heat shock, and starvation, we tested their genoprotective properties. Therefore, we treated flies by investigated drugs with following acute exposure by γ-radiation at the dose 1000 Gy. Quercetin, (-)-epicatechin, and ibuprofen at the concentration 1.0 μM increased the survival of males and females by 19–51% on average (*p* < 0.05; Figure 6). The most radioprotective effect was found for flavonoid (-)-epicatechin.

Thus, we have found that (-)-epicatechin and ibuprofen have the significant protective effect against the oxidative (paraquat) and genotoxic (γ-radiation) stresses. Quercetin treatment led to radioprotection in males only without significant positive effects on stress resistance to other types of stresses.

### The activation of stress-response genes after quercetin, (-)-epicatechin, and ibuprofen treatment

**Figure 7:**
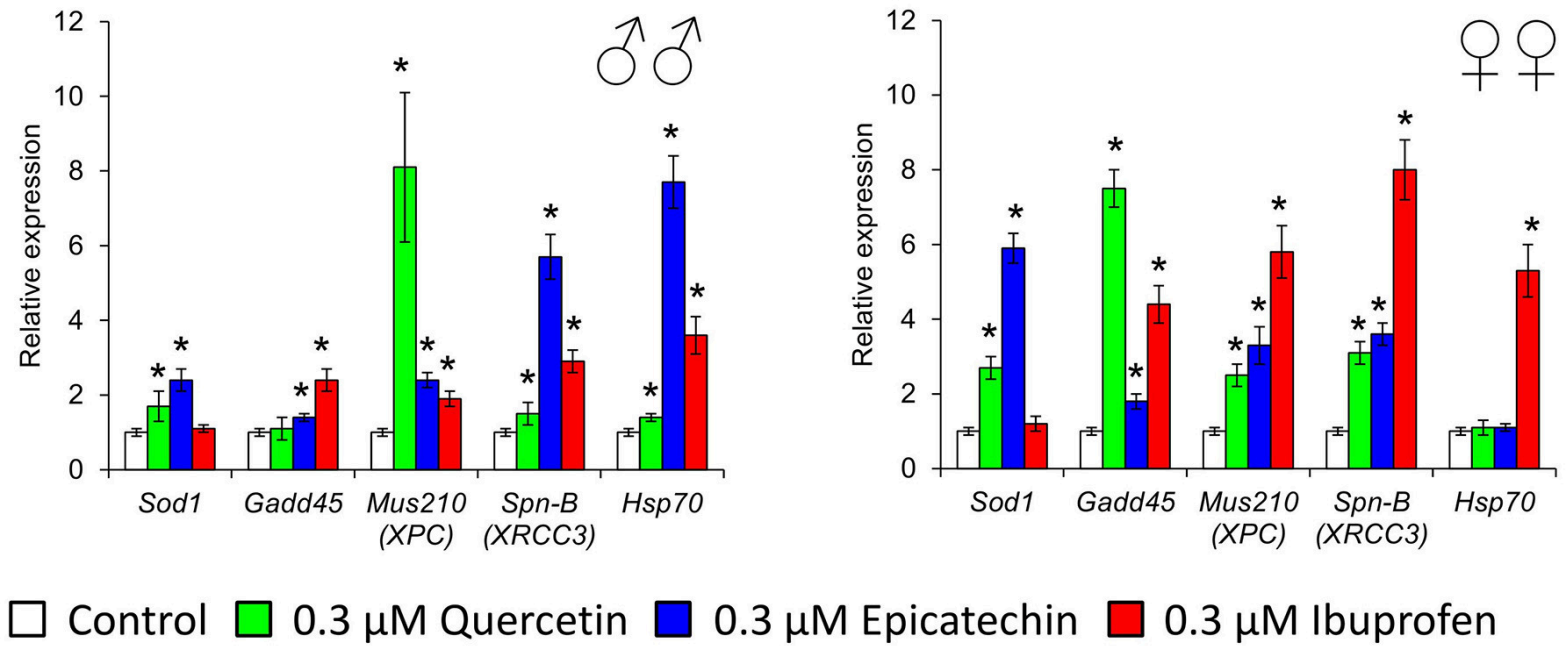
Expression of stress-response genes in ***Drosophila melanogaster*** males (♂♂) and females (♀♀) treated with quercetin, (-)-epicatechin, and ibuprofen; ^*^p < 0.05, Mann-Whitney *U*-test.

To investigate some molecular mechanisms of effects of quercetin, (-)-epicatechin, and ibuprofen on the *Drosophila* lifespan and stress resistance, we analyzed the expression of stress-response genes that provide detoxification of free radicals (*Sod1*), heat shock response (*Hsp70*), coordination of the DNA damage response (*Gadd45*), DNA excision repair (*Mus210*), and double-strand break repair (*Spn-B*). Quercetin stimulated the activity of *Sod1, Mus210, Spn-B* in males and females, and *Gadd45* in females by 1.5–8.1 times (*p* < 0.05; Figure 7). Increased mRNA levels of *Sod1, Gadd45, Mus210, Spn-B* genes in males and females, and *Hsp70* in males by 1.4–7.7 times (*p* < 0.05) were also detected after the treatment with (-)-epicatechin (Figure 7). Ibuprofen sufficiently enhanced the activity of DNA repair genes and *Hsp70* by 1.9–8.0 times (*p* < 0.05) without the effect to the expression of *Sod1* (Figure 7).

## Discussion

Our results complement data obtained by other authors regarding dose-dependent effects of quercetin, (-)-epicatechin, and ibuprofen. In our studies the long-term consumption of quercetin and (-)-epicatechin at concentrations 0.3–1.0 μM had no positive or in some cases even negative effects on the lifespan of *Drosophila melanogaster*. In contrast, the treatment with ibuprofen at same concentrations extended the lifespan of fruit flies (He et al., 2014). At the same time, the short-term consumption of (-)-epicatechin resulted in positive effects on lifespan parameters of both sexes when it was applied after hatching (at the age of 0–10 days), and in females after the consumption at the age of 30–40 days. The short-term consumption of quercetin at the young and middle age led to the increase of lifespan of females and had no effects on males. However, the treatment during 10 days with ibuprofen extended the lifespan of females only when it was carried out from the age of 30 days. It must be noted that the positive effect of the short and delayed ibuprofen application was more significant than the action of the long-term treatment (6–17% compared with 23%). Control of age-dependent physiological parameters (fecundity and locomotor activity) demonstrated the neutral or positive action of investigated drugs at concentrations 0.3–1.0 μM on the life quality of females. However, the physical activity males were suppressed by almost all variants of pharmacological treatments.

Effects of both flavonoids were previously studied on different model organisms. However, the results were controversial. For example, one study showed that quercetin increases the lifespan of *Saccharomyces cerevisiae* (Belinha et al., 2007). In another one quercetin failed to the prolong lifespan of yeast (Howitz et al., 2003). The possible explanation of the failure given by the authors is the compound oxidation or the insufficient cellular uptake. Quercetin also failed to increase the lifespan of LACA mice (Jones and Hughes, 1982) and F1 hybrid mice (Spindler et al., 2013). Numerous studies demonstrated that quercetin increases the lifespan of *Caenorhabditis elegans*, but underlying mechanisms are not fully determined (Kampkotter et al., 2008; Saul et al., 2008; Pietsch et al., 2009). Effects of pure (-)-epicatechin on the lifespan of different model organisms were also studied. In numerous studies (-)-epicatechin failed to prolong lifespan of worms (Sunagawa et al., 2011; Surco-Laos et al., 2012). However, in concentrations 0.01–1 mg/ml (approximately 0.035–3.5 mM) it increased the median lifespan of *Drosophila melanogaster* males by 1.8–7.4% (Massie et al., 1993). Additionally, there are data that (-)-epicatechin also increased the lifespan of diabetic mice (Si et al., 2011).

**Figure 8:**
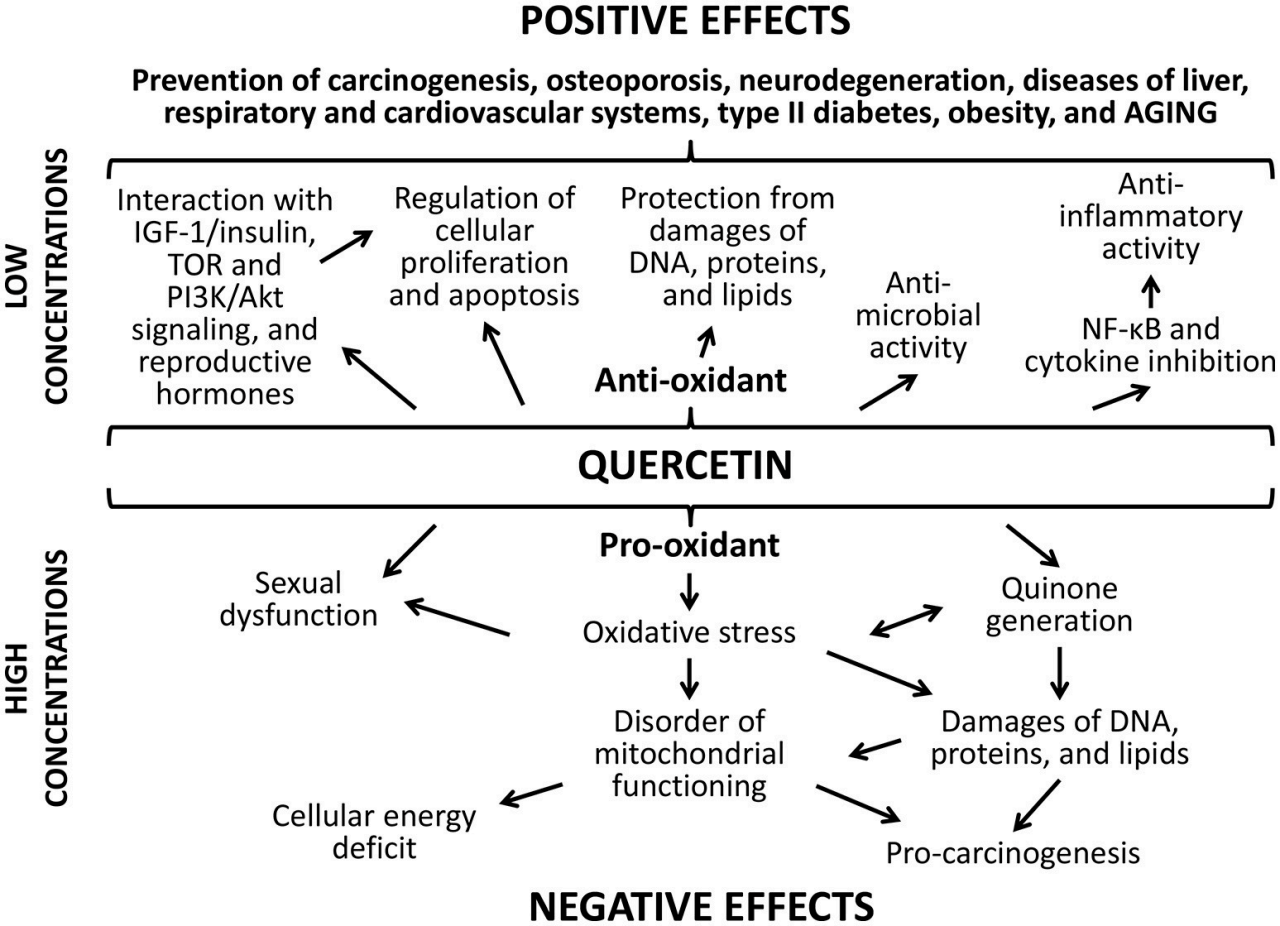
**Positive and negative effects of quercetine**.

**Figure 9:**
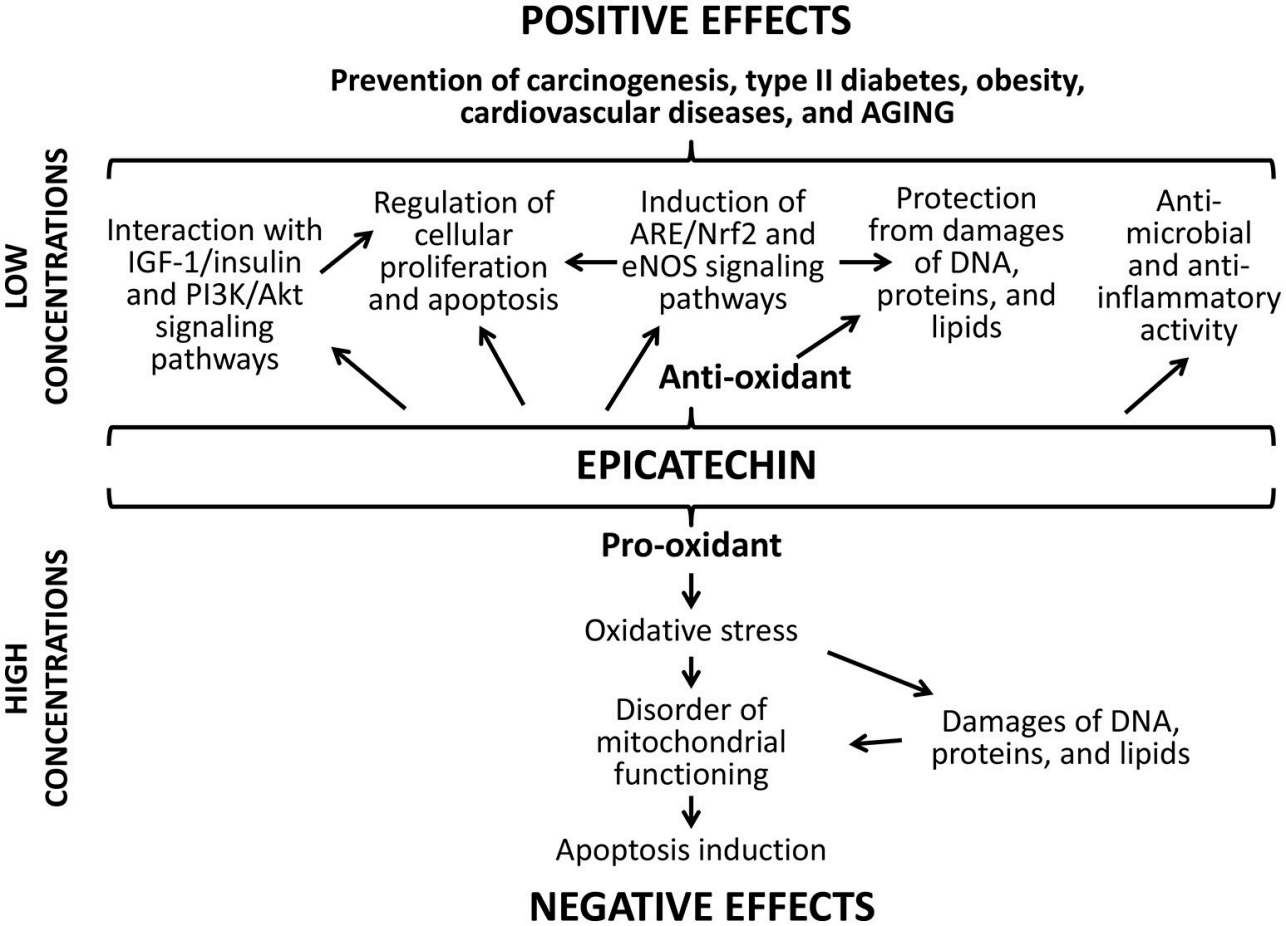
**Positive and negative effects of (-)-epicatechin**.

The positive action of studied flavonoids can be a result of numerous underlying mechanisms (Figures 8, 9). Firstly, well-known anti-oxidant properties of quercetin and (-)-epicatechin provide the protection of cellular macromolecules from damages and predict the mitochondrial dysfunction (Khan et al., 2016). In this way, studied drugs demonstrated benefit action against the development of aging-related pathologies including neurodegeneration (Shay et al., 2015; Barreca et al., 2016; Elumalai and Lakshmi, 2016), cardiovascular and liver diseases (Ansar et al., 2016; Cheserek et al., 2016), diabetes (Shay et al., 2015; Haddad and Eid, 2016), cancer (Shay et al., 2015; Khan et al., 2016).

Secondly, flavonoids can directly affect multiple molecular pathways, which regulate lifespan in different organisms. The free-radical scavenging activity of quercetin and (-)-epicatechin is complemented by the nucleus translocation of the Nrf2 transcription factor, where it binds to antioxidant response element (ARE) and promotes expression of detoxifying and antioxidant enzymes (Granado-Serrano et al., 2010; Si et al., 2011; Moreno-Ulloa et al., 2015; Li et al., 2016). In our experiments, the activation of transcription of the Nrf2 target gene *Sod1* after the treatment with quercetin and (-)-epicatechin was observed. At the same time, flavonoids regulate the activity of other signaling pathways that determined their non-antioxidant activity. For example, quercetin and (-)-epicatechin are able to modulate activity of pro-aging IGF-1/insulin and phosphatidylinositol-3-kinase/protein kinase B (PI3K/Akt) pathways (Pietsch et al., 2012) (Si et al., 2011). Further, IGF-1/insulin suppression leads to translocation of the FOXO transcription factor to nucleus and activation of expression of pro-longevity genes (Si et al., 2011). In the present study, we found activation of the target FOXO gene *Gadd45* with known geroprotective activity (Moskalev et al., 2012; Plyusnina et al., 2012). Quercetin also inhibits target of rapamicin (mTOR) (Granado-Serrano et al., 2006; Meng et al., 2015). Its inactivation influences the aging rate through the induction of autophagy and the repressing of protein synthesis and cell growth (Johnson et al., 2013). Additionally, suppression of TOR and IGF-1/insulin signaling pathways leads to enhanced longevity of model organisms through the activation of heat shock factor 1 (HSF-1) (Seo et al., 2013). We obtained the activation of its target *Hsp70* in *Drosophila* males after the treatment with (-)-epicatechin and quercetin. Both flavonoids regulate the transcription factor NF-κB, a regulator of cytokines and growth factors production, and thus protects against chronic inflammation (Nair et al., 2006; Vicentini et al., 2011). Thus, studies on different model organisms showed that quercetin and (-)-epicatechin can induce several pro-longevity mechanisms and inhibit aging processes.

Thirdly, the increase of the *Drosophila* lifespan after the short-term treatment with quercetin and (-)-epicatechin, as well as their properties to stimulate stress resistance and some physiological parameters can be a result of hormetic response (Son et al., 2008). It was shown, that both drugs are able to produce oxidative stress and damage DNA (Yamashita and Kawanishi, 2000; Oikawa et al., 2003). Thus, flavonoids can influence as a mild stress and activate protection mechanisms in cells. It was shown by other authors that effects of quercetin on the lifespan of nematodes have common feateres with the hormesis dose-dependent response curve (Pietsch et al., 2011). In our experiments, the short-term consumption of studied flavonoids induced expression of genes provided the free radical detoxification (*Sod1*), heat shock response (*Hsp70*), DNA damage response and repair (homologs of *Gadd45, XPC, XRCC3*). This effect can be a manifestation of the compensator hormetic response to the pharmacological treatment.

Detected negative effects of the long-term flavonoids' application can be a visualization of their toxic properties, which quercetin and (-)-epicatechin possess aside from health-benefiting effects. For example, quercetin, when it protects against free radicals, is converted into the toxic product, which can damages proteins through the reactivity toward thiols (Boots et al., 2008). This effect is known as quercetin paradox (Boots et al., 2007). Additionally, flavonoides can balance the hormonal background. It was shown that quercetin can regulate the production of steroidal hormones and predicts some pathological processes (Bharti et al., 2014; Shahzad et al., 2014). However, high concentrations of quercetin have the negative influence on sexual function, for example, it potentially can cause the disruption of follicular development (Santini et al., 2009) and induce the uterus neoplastic process in rodents (Shahzad et al., 2014). Nevertheless, we didn't found negative effect of flavonoids to the reproductive function of *Drosophila* females.

**Figure 10:**
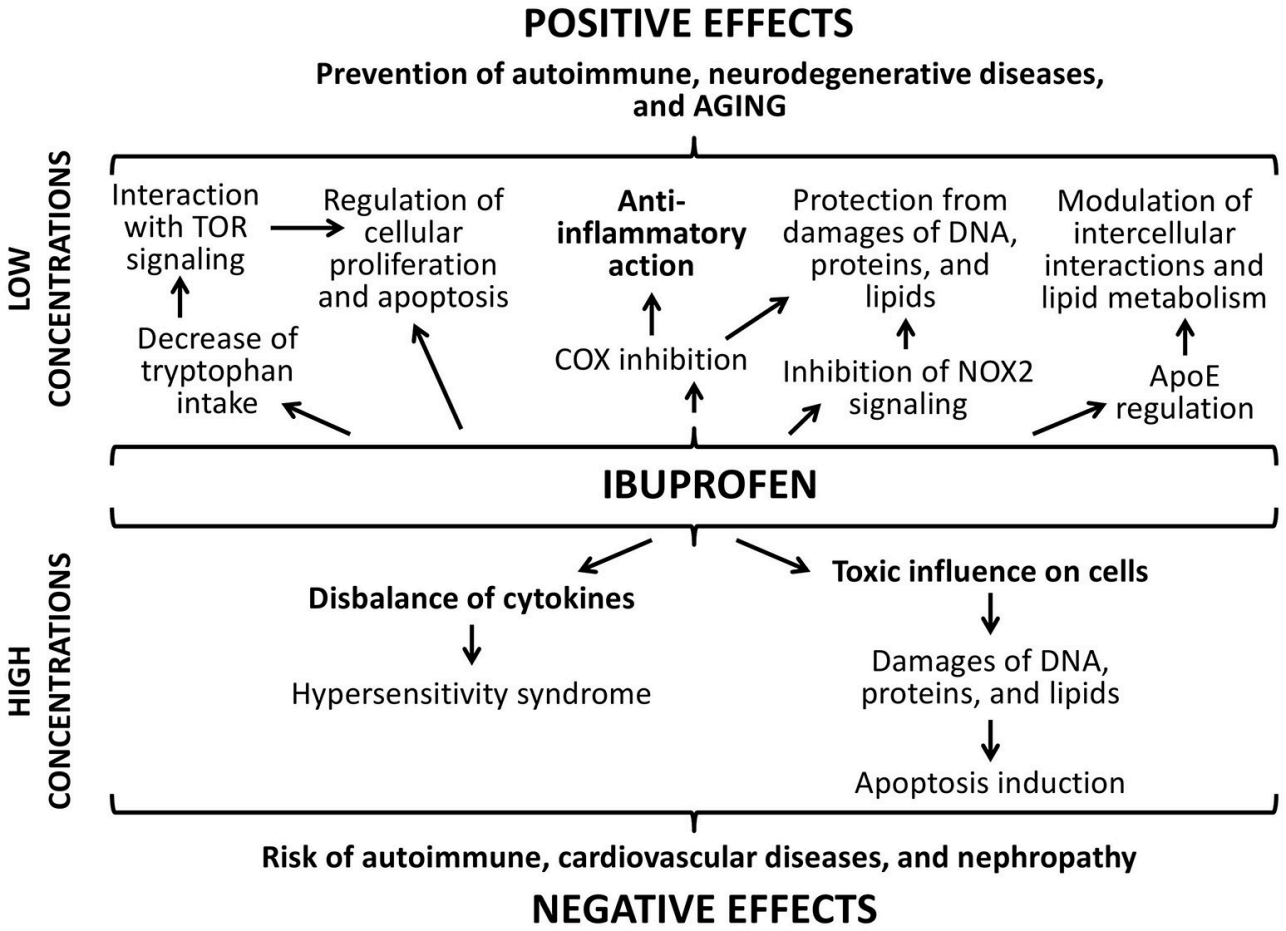
**Positive and negative effects of ibuprofen**.

Effects of ibuprofen are dose-dependent as well (Figure 10). The main positive effect ibuprofen is its ability to reduce the inflammation by inhibiting cyclooxygenase (COX) (Smith et al., 2000). It also suppresses oxidative stress through the inhibition of the NADPH oxidase (NOX2) that was demonstrated in brains of mice with Alzheimer's disease (Wilkinson et al., 2012). However, one of the central mechanisms that increase the lifespan in yeast, nematodes, and flies under the ibuprofen treatment is its ability to reduce tryptophan import into cells (He et al., 2014). Through the mediation of the tryptophan metabolism, ibuprofen can suppresses aging-associated signaling pathways, for example, TOR signaling. Hyperfunction of such mechanisms develops during the growth of an adult organism and contributes age-related pathologic processes (Blagosklonny and Hall, 2009; Johnson et al., 2013). Their inhibition at the middle age using ibuprofen can cause the pronounced life extending effect that we observed in the present study. Additionally, ibuprofen can regulate the activity of the protein apolipoprotein E that associated with the neurodegenerative processes in mammalian brain, including development of Alzheimer's and Parkinson's diseases, through the COX-2 inhibition and PPAR-γ activation (Melton et al., 2003; Lichtenstein et al., 2010; DiBattista et al., 2016). Investigations on transgenic fruit flies demonstrated that this effect can influence the lifespan and aging rate (Lichtenstein et al., 2010).

However, the consumption of ibuprofen may lead to the development of different hypersensitivity reactions due to the disbalance of activity of cytokines and immunoglobulins (Nanau and Neuman, 2010; Blanca-Lopez et al., 2015). Ibuprofen can cause the enhanced generation of reactive oxygen species and leads to DNA damages, protein carboxylation, and lipid peroxidation (Gómez-Oliván et al., 2014; Husain et al., 2015). Ibuprofen decreases quality of sperm in mice and its chromatin/DNA integrity (Roodbari et al., 2015). As a compensator reaction, in the cells the activity of antioxidant enzymes is induced. However, we didn't found the activation of *Sod1* after the treatment with ibuprofen at concentration 0.3 μM that we used. At the same time, we found the significant increase of the transcriptional activity of genes provided DNA damage response and repair (homologs of *Gadd45, XPC, XRCC3*), and protein structure maintenance (*Hsp70*) that can be a result of both stimulation of cellular protective pathways and hormetic response.

In our study, quercetin, (-)-epicatechin, and ibuprofen increased the resistance of flies to acute γ-irradiation. The more pronounced effects were obtained for (-)-epicatechin. These results are corresponded to results obtained by other authors. It was shown that (-)-epicatechin can increase the survival of fibroblasts after radiation by restoring mitochondrial membrane potential, inhibiting reactive oxygen species generation and MAPK activity (Shin et al., 2014). In the same study, the ability of the compound to provide the protection against radiation *in vivo* was revealed in the experiment with zebrafish embryos. Both studied flavonoids are able to protect DNA from damage caused by ionizing radiation exposure (Nair and Salvi, 2008; Özyurt et al., 2014). At the same time, it was shown that ibuprofen has minor protective effects against radiation in mice (Gross et al., 1991). In our experiments, (-)-epicatechin and ibuprofen increased the resistance to oxidative stress of *Drosophila melanogaster* flies. In other study, the addition of (-)-epicatechin didn't increase the *Drosophila* resistance to paraquat (Kim et al., 1997). The possible explanation is differences in time of flies' exposure to flavonoid and differences in concentrations of paraquat and (-)-epicatechin. It must be noted that ibuprofen demonstrated the most significant antioxidant effect. Previously minor data demonstrated that this anti-inflammatory drug can exercise antioxidant properties predominantly through the COX inhibition (Milatovic et al., 2011; Zaminelli et al., 2014). However, this mechanism is absented in flies. At the same time we found the sufficient activation of DNA repair and heat shock response genes.

Our data support an idea that quercetin and (-)-epicatechin consumption in proper low doses can increase life span of *Drosophila melanogaster*. Furthermore, it can increase the resistance of flies to oxidative stress and ionizing radiation. The possible explanation of observed effects is the hormetic response. Ibuprofen can also stimulate cellular protective mechanisms and induces the resistance of the organism to oxidative and genotoxic stress. Obtained results demonstrate the high potential of the using of these chemicals as geroprotective and adaptogenic drugs.

## Author contributions

EP, EL, AK, MS, and AM wrote the manuscript text. EP, ED, NZ, and MS carried out the experiments and processed the statistical analysis. AM supervised the research and the text of the manuscript. All authors read and approved the final manuscript.

## Funding

This work was supported by the Grant of the Russian Science Foundation N○ 14-50-00060.

### Conflict of interest statement

The authors declare that the research was conducted in the absence of any commercial or financial relationships that could be construed as a potential conflict of interest.
